# Proteomic Analysis of Preoperative CSF Reveals Risk Biomarkers of Postoperative Delirium

**DOI:** 10.3389/fpsyt.2020.00170

**Published:** 2020-03-04

**Authors:** Yongzheng Han, Wei Chen, Yanan Song, Yi Yuan, Zhengqian Li, Yang Zhou, Taotao Liu, Dengyang Han, Xinning Mi, Min Li, Geng Wang, Lijun Zhong, Juntuo Zhou, Xiangyang Guo

**Affiliations:** ^1^Department of Anesthesiology, Peking University Third Hospital, Beijing, China; ^2^Center of Medical and Health Analysis, Peking University Health Science Center, Beijing, China; ^3^Department of Anesthesiology, Beijing Jishuitan Hospital, Beijing, China; ^4^Beijing Advanced Innovation Center for Big Data-Based Precision Medicine, Beihang University, Beijing, China

**Keywords:** cerebrospinal fluid, postoperative delirium, hip fracture, protein profile analysis, older patients

## Abstract

**Objective:** To analyze the proteome of preoperative cerebrospinal fluid (CSF) in older orthopedic patients with or without postoperative delirium (POD) using untargeted proteomics.

**Methods:** A prospective cohort study was conducted. Eighty hip fracture patients aged ≥65 years were recruited. After successful spinal anesthesia, CSF was collected. The patients were divided into POD and No-POD groups based on the Confusion Assessment Method, and patients with POD were graded using the Memorial Delirium Assessment Scale (MDAS). Thirty No-POD patients were matched to 10 POD patients by age (±2 years) and Mini–Mental State Examination score (±2 scores). Label-free proteomic analysis was performed using a liquid chromatography coupled to mass spectrometry (LC-MS) workflow. Validation was performed using mass-spectrometry-based parallel reaction monitoring (PRM) for the 30 No-POD and 10 POD patients, as well as for an additional 5 POD patients. Bioinformatics were used to investigate possible relevant pathological mechanisms.

**Results:** The incidence of POD in older orthopedic patients was 18.8% in our cohort of 80 patients. Proteomics results revealed 63 dysregulated CSF proteins, and PRM analysis validated these results. The preoperative CSF levels of both V-set and transmembrane domain-containing protein 2B (VSTM2B) and coagulation factor V (FA5) were positively correlated with MDAS scores on postoperative day 1 (*r* > 0.8, *p* < 0.05). Bioinformatic analysis revealed that several nervous-system-related pathways are relevant to POD development.

**Conclusion:** We identified and validated several novel CSF proteins that are dysregulated in POD, and revealed several pathways that are relevant to POD development. Our results not only provide risk biomarkers for POD, but also give clues for further investigations into the pathological mechanisms of delirium.

**Clinical trial registration:** This study was registered in the Chinese Clinical Trial Registry (ChiCTR1900021533).

## Introduction

Postoperative delirium (POD) is an acute neuropsychiatric syndrome characterized by cognitive dysfunction and decreased attention after anesthesia and surgery (1). Preoperative brain function reserve is often low in patients at high risk of POD. POD is a common complication following surgery for hip fracture induced by traumatic stimulation in older patients, with an incidence of 4.0–53.3% (2). With an aging population and an increase in older patients undergoing surgery, the incidence of POD has increased significantly (3). Epidemiological investigation has revealed that POD occurs mainly 24–72 h after surgery, increases mortality by 10–20%, and has a serious adverse effect on patient prognosis, including an increased risk of cognitive impairment and the development of Alzheimer's disease (4, 5). With the acceleration of aging in the global population, POD incidence has become one of the main indicators of medical quality (6).

Because the blood–brain barrier tightly regulates the flow of molecules between the brain and the vasculature, cerebrospinal fluid (CSF) is the most likely body fluid to accurately reflect any biochemical changes that occur following damage to the central nervous system. The pathogenesis of POD is unclear, and clinical symptoms in patients are diverse and often fluctuate; thus, the identification of specific biomarkers to predict and diagnose POD is a major clinical problem that urgently needs solving. Previous research has revealed that hip fracture patients who developed POD had lower preoperative CSF concentrations of anti-inflammatory cytokines (7). Evaluating imbalanced protein expressions in the CSF using protein spectrum analysis may allow us to better understand the pathological changes that occur at the molecular level in POD. The early detection of patients at high risk of POD will optimize perioperative management and promote rapid recovery after surgery.

We hypothesized that CSF proteins that were differentially expressed at the preoperative stage would be associated with POD severity in older orthopedic patients. The findings from the current study may encourage more studies to investigate the roles of proteins in POD neuropathogenesis, and may also facilitate more protein biomarker studies in POD.

## Materials and Methods

This study was performed in accordance with the Declaration of Helsinki and was approved by the Beijing Jishuitan Hospital Medical Science Research Ethics Committee (JLKS201901-04). We performed a prospective cohort study with written informed consent, which was registered at the Chinese Clinical Trial Registry (ChiCTR1900021533).

### Study Population

**Figure 1:**
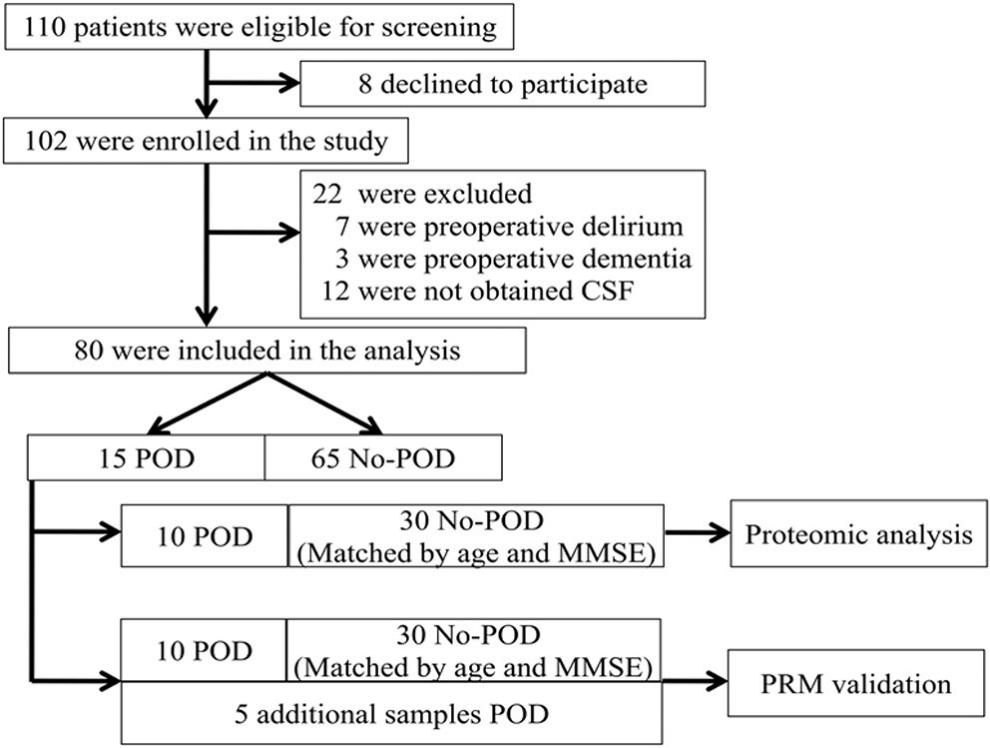
Flow diagram showing the recruiting criterion. One hundred and ten patients were initially screened for the study, and 80 patients were finally included in the data analysis. CSF, cerebrospinal fluid; POD, postoperative delirium; MMSE, mini-mental state examination; PRM, parallel reaction monitoring.

The study was conducted at Beijing Jishuitan Hospital (Beijing, China) between March 2019 and August 2019. Eligible patients were at least 65 years old, had an acute hip fracture injury (within 3 days), had an American Society of Anesthesiologists (ASA) physical class of I–III, and were scheduled to have a hip internal fixation or hip arthroplasty under spinal anesthesia. A total of 110 adults were invited to participate in this study (Figure 1, flow diagram). After reviewing patient medical records, patients were excluded if they (1) had a past medical history of neurological disease or a clinically evident neurovascular disease (e.g., delirium, schizophrenia, dementia, or stroke). Dementia was defined as a Mini–Mental State Examination (MMSE) score of ≤17 for illiterate patients, ≤20 for patients with 1–6 years of education, and ≤24 for patients with 7 or more years of education (8); (2) were unable to read or had severe visual or auditory deficits; (3) had a history of alcohol abuse and drug dependence; or (4) were unwilling to comply with the study protocol or procedures. We selected 10 POD patients at random, using a random number table, for proteomic analysis. Next, patients without POD (no-POD patients; 30 cases) were matched to the selected POD patients by age (±2 years) and MMSE score (±2 points). After admission, all patients stopped taking the drugs before, and accepted oral Oxycodone/Acetaminophen (5 mg/325 mg, four times a day) given by the ward to relieve pain.

### Neuropsychological Testing

We interviewed all patients the day before surgery and the MMSE was performed. The Confusion Assessment Method (CAM) was used to identify patients who experienced preoperative delirium. The CAM was performed twice daily on the first and second days after surgery (8:00 and 20:00), which is the period in which POD is usually diagnosed after hip fracture surgery in older adult patients (9). The presence or absence of POD was defined according to CAM results, and the severity of POD was defined according to the Memorial Delirium Assessment Scale (MDAS) (10). The Chinese versions of the CAM and MDAS have good reliability and validity in the older Chinese population (11).

### Anesthesia and Surgery

All participants underwent hip internal fixation or hip arthroplasty under spinal anesthesia. All surgeries were performed by the same surgical team to avoid potential confounding factors caused by varying surgical skills or different surgical practices. Electrocardiography, pulse oximetry, and non-invasive blood pressure were all continuously monitored during anesthesia and were recorded at fixed intervals of 3 min. Postoperatively, all patients received intravenous patient-controlled analgesia with the same analgesic regimen (sufentanil 100 μg and ondansetron 8 mg, in 100 mL normal saline).

### Sample Collection

The CSF (2 mL) was collected in a polypropylene tube during spinal anesthesia, prior to the administration of local anesthetic. Samples were centrifuged immediately at 3,000 rpm for 10 min at 4°C to remove cells (12), and the supernatant was aliquoted and stored at −80°C until needed.

### CSF Protein Digestion

CSF samples (300 μL) from each patient were processed according to the manufacturer's protocol for filter-aided sample preparation (13). For each filter, 1 μg of trypsin (Promega, Madison, WI, USA) in 100 μL of 50 mM NH_4_HCO_3_ was added, with a protein-to-enzyme ratio of approximately 50:1. Samples were incubated overnight at 37°C and released peptides were collected by centrifugation.

### Liquid Chromatography Tandem-Mass Spectrometry (LC-MS/MS) Analyses

The MS analysis experiments were performed on a nano-flow high pressure liquid chromatography (HPLC) system (U3000 UHPLC, ThermoFisher Scientific, Waltham, MA, USA) connected to a Q-Exactive HF Mass Spectrometer (ThermoFisher Scientific) equipped with a Nanospray Flex Ion Source (ThermoFisher Scientific). Chromatographic separation was performed on a homemade reversed-phase C18 column (3 μm particles, 75 μm internal diameter × 250 mm) at a flow rate of 300 nL/min with a 150-min gradient of 5–35% acetonitrile in 0.1% formic acid. The electrospray voltage was maintained at 2.2 kV and the capillary temperature was set at 320°C. The Q-Exactive HF Mass Spectrometer was operated in data-dependent mode. All samples were analyzed in random order.

### Protein Identification and Quantification

Raw MS data processing was performed using MaxQuant software (version 1.4.1.2, http://www.maxquant.org/). For protein identification, the MS/MS data were submitted to the Uniprot human protein database (release 3.43; 72 340 sequences) using the Andromeda search engine with the following settings: trypsin cleavage; fixed modification of carbamidomethylation of cysteine; variable modifications of methionine oxidation; a maximum of two missed cleavages. The false discovery rate was calculated by decoy database searching, and a false discovery rate of 0.01 at both the peptide and protein levels was used as the cutoff. Other parameters were set as default. Label-free quantitation (LFQ) was performed in MaxQuant. The minimum ratio count for LFQ was set to 2, and the match-between-runs option was enabled with retention time tolerance of 2 min. Other parameters were set as default. Upregulated or downregulated proteins were defined as those with a significantly changed protein ratio (*p* < 0.05, student's *t*-test). The *p*-values were calculated using Perseus software (version 1.6.6.0, http://www.perseus-framework.org/).

### Parallel Reaction Monitoring (PRM) Validation of Dysregulated Proteins

Considering that most commercially available ELISA kits are developed for blood and urine samples rather than CSF samples, because of the low protein concentrations in CSF, we used an LC-MS-based PRM technique to validate the untargeted proteomic results. The high throughput in PRM analysis makes it more efficient than ELISA to use in a validation study. PRM method construction, optimization, and data processing were performed using Skyline software. For method construction and optimization, untargeted proteomic results were used as a reference for the peptide and transition selections. Targeted proteins were filtered based on the following criteria: (1) at least two unique peptides were detected; (2) at least five product ions were detected for each peptide; and (3) no post-translational modifications were observed in the peptide. After method construction and optimization, 20 proteins were selected for a final validation assay using the discovery sample cohort (used for untargeted proteomic analysis, and made up of 10 POD samples and 30 No-POD samples) and an additional 5 POD samples.

PRM analysis was performed on a nano-LC-MS system in the same way as the untargeted proteomic analysis was performed. The chromatographic separation was performed on a homemade reversed-phase C18 column (3 μm particles, 75 μm I.D. × 250 mm) at a flow rate of 300 nL/min with a 75-min gradient of 5–35% acetonitrile in 0.1% formic acid. The electrospray voltage was maintained at 2.2 kV, and the capillary temperature was set at 320°C. The Q-Exactive HF Mass Spectrometer was operated in PRM mode. All samples were analyzed in random order. After MS data acquisition, raw data were imported into Skyline and MS2-based quantification (peak areas of extracted ion chromatography of 3–5 fragment ions were calculated and used for quantification) was performed.

### Bioinformatic Analyses

Statistical analysis for the untargeted proteomics data was performed using Perseus software (version 1.6.6.0) (14). A hierarchical clustering-based heat map was drawn using the MetaboAnalyst web server (15) (https://www.metaboanalyst.ca/). Protein–protein interaction networks and Gene Ontology (GO) enrichment analyses were performed using STRING (16). The dysregulated proteins revealed by untargeted proteomics were used as the input. Significantly enriched pathways were retrieved by searching against the REACTOME database.

### Statistical Analysis

The data are expressed as the mean ± standard deviation (SD), the median and interquartile range (IQR), or the number (%). The Kolmogorov–Smirnov method was used to test the normality of all of the variables. Categorical variables were analyzed using a χ^2^ test, while continuous variables were analyzed using an independent-samples *t*-test. The Mann–Whitney *U*-test was used to analyze non-normal variables. Statistical significance was set at *p* < 0.05. SPSS software (version 21.0; IBM Corp., Armonk, NY, USA) and Perseus software (version 1.6.6.0) were used for data analysis.

## Results

### Participant Characteristics

Subject characteristics are shown in Table 1. Of the 80 patients, 15 had POD and 65 did not have POD; the incidence of POD in our cohort of older orthopedic patients was 18.8%. In the discovery cohort, there were no differences in age, MMSE score, sex, height, weight, body mass index (BMI), ASA class, education years, length of anesthesia and surgery, Charlson comorbidity score and preoperative visual analog scale (VAS) between the POD and No-POD groups. The length of anesthesia was defined as being from the time that the anesthesiologists started spinal anesthesia in the patients to the time when the patients were sent to the post-anesthesia care unit. The length of surgery was defined as being from the time of the initial incision to the time of skin closure.

**Table 1:** Subject characteristics.

	**POD group (*n* = 10)**	**No-POD group (*n* = 30)**	**Statistical test**	***P*-value**
Age (years), mean ± SD	82.2 ± 7.6	81.7 ± 7.2	*t* = 0.188	0.852
MMSE score, mean ± SD	25.2 ± 3.9	25.4 ± 3.6	*t* = −0.174	0.863
Male, *n* (%)	5 (50.0)	7 (23.3)	χ^2^=1.429	0.232
Height (cm), mean ± SD	165.0 ± 7.9	163.2 ± 9.4	*t* = 0.516	0.609
Weight (kg), mean ± SD	66.1 ± 14.2	63.1 ± 10.5	*t* = 0.647	0.522
BMI (kg/m^2^), mean ± SD	24.6 ± 5.4	23.3 ± 3.0	*t* = 0.846	0.404
ASA class, *n* (%)			χ^2^=0.342	0.559
II	8 (80.0)	19 (63.3)		
III	2 (20.0)	11 (36.7)		
Education (years), median (IRQ)	13.5 (7.8)	9.0 (9.8)	z = −0.793	0.428
Length of anesthesia (min), mean ±SD	91.9 ± 12.5	93.8 ± 30.7	*t =* −0.171	0.865
Length of surgery (min), mean ±SD	63.8 ± 11.6	75.8 ± 33.4	*t*=-0.994	0.328
Charlson Comorbidity score, mean ±SD	5.9 ± 1.0	6.3 ± 1.7	*t* = −0.671	0.506
Preoperative VAS score, mean ±SD	3.6 ± 1.0	3.2 ± 0.9	*t* = 0.786	0.441

*POD, postoperative delirium; MMSE, Mini-Mental State Examination; ASA, American Society of Anesthesiologists; IQR, interquartile range; VAS, Visual Analog Scale; cm, centimeter; kg, kilogram; min, minute; SD, standard deviation*.

### Untargeted Proteomics

**Figure 2:**
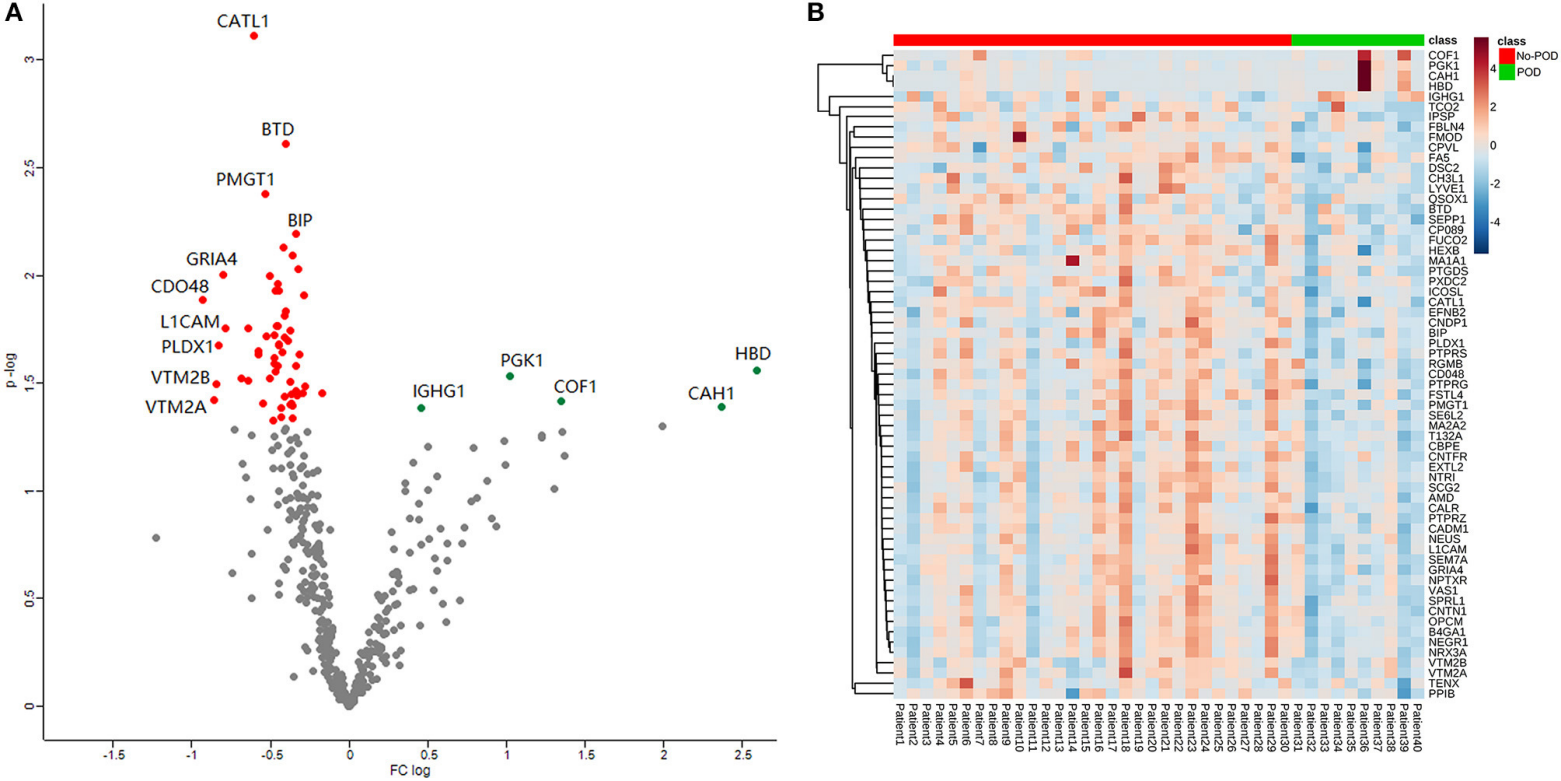
Volcano plot and heat map. Volcano plot showed the distribution of the 412 quantitative proteins in terms of fold change and *P*-value **(A)**. Heat map showed the expression profile of the 63 dysregulated proteins **(B)**.

A total of 1,076 proteins were identified, and after filtration (the detection ratio was over 80% in both groups), 412 remained in the search for dysregulated proteins. After statistical analysis, 63 proteins were revealed to be significantly different between the POD and No-POD groups (*p* < 0.05; Table S1). A volcano plot shows the distribution of the 412 quantitative proteins in terms of fold change and *p*-values (Figure 2A); of these proteins, five were upregulated and 58 were downregulated in the POD group. A heat map shows the expression profiles of the 63 dysregulated proteins (Figure 2B).

### Validation Using PRM

In the PRM-based validation assay, 20 dysregulated proteins were quantified in a group of 45 patients, made up of the discovery sample cohort (*n* = 40) and an additional 5 samples with POD. Results from the statistical analysis are presented in terms of fold change and *t*-test *p*-values (Table 2). All 20 proteins were downregulated in the POD group, consistent with the trends observed in the untargeted proteomic analysis.

**Table 2:** Quantitative results of the PRM assay.

**Protein**	**Fold change result**	**Adjusted *P*-value**
sp|Q8TAG5|VSTM2A_HUMAN	0.62 (95% CI:0.47 to 0.81)	0.0183
sp|P48058|GRIA4_HUMAN	0.58 (95% CI:0.41 to 0.8)	0.0183
sp|P07711|CATL1_HUMAN	0.62 (95% CI:0.44 to 0.88)	0.0403
sp|O95502|NPTXR_HUMAN	0.53 (95% CI:0.36 to 0.78)	0.0183
sp|Q7Z3B1|NEGR1_HUMAN	0.65 (95% CI:0.47 to 0.9)	0.0403
sp|Q15904|VAS1_HUMAN	0.75 (95% CI:0.57 to 0.98)	0.0806
sp|Q9Y4C0|NRX3A_HUMAN	0.58 (95% CI:0.4 to 0.83)	0.0287
sp|Q9BY67|CADM1_HUMAN	0.62 (95% CI:0.46 to 0.83)	0.0183
sp|O75144|ICOSL_HUMAN	0.66 (95% CI:0.49 to 0.89)	0.0403
sp|O43505|B4GA1_HUMAN	0.71 (95% CI:0.57 to 0.9)	0.0309
sp|Q99574|NEUS_HUMAN	0.62 (95% CI:0.45 to 0.85)	0.0287
sp|P33908|MA1A1_HUMAN	0.66 (95% CI:0.49 to 0.89)	0.0403
sp|P36222|CH3L1_HUMAN	0.68 (95% CI:0.51 to 0.91)	0.0403
sp|P43251|BTD_HUMAN	0.68 (95% CI:0.54 to 0.86)	0.0183
sp|P16870|CBPE_HUMAN	0.68 (95% CI:0.51 to 0.91)	0.0403
sp|Q14515|SPRL1_HUMAN	0.69 (95% CI:0.57 to 0.85)	0.0183
sp|Q6UX73|CP089_HUMAN	0.74 (95% CI:0.59 to 0.93)	0.0403
sp|Q96KN2|CNDP1_HUMAN	0.73 (95% CI:0.57 to 0.92)	0.0403
sp|Q12860|CNTN1_HUMAN	0.67 (95% CI:0.52 to 0.88)	0.0287
sp|P41222|PTGDS_HUMAN	0.62 (95% CI:0.44 to 0.89)	0.0403

### Bioinformatic Analyses

**Figure 3:**
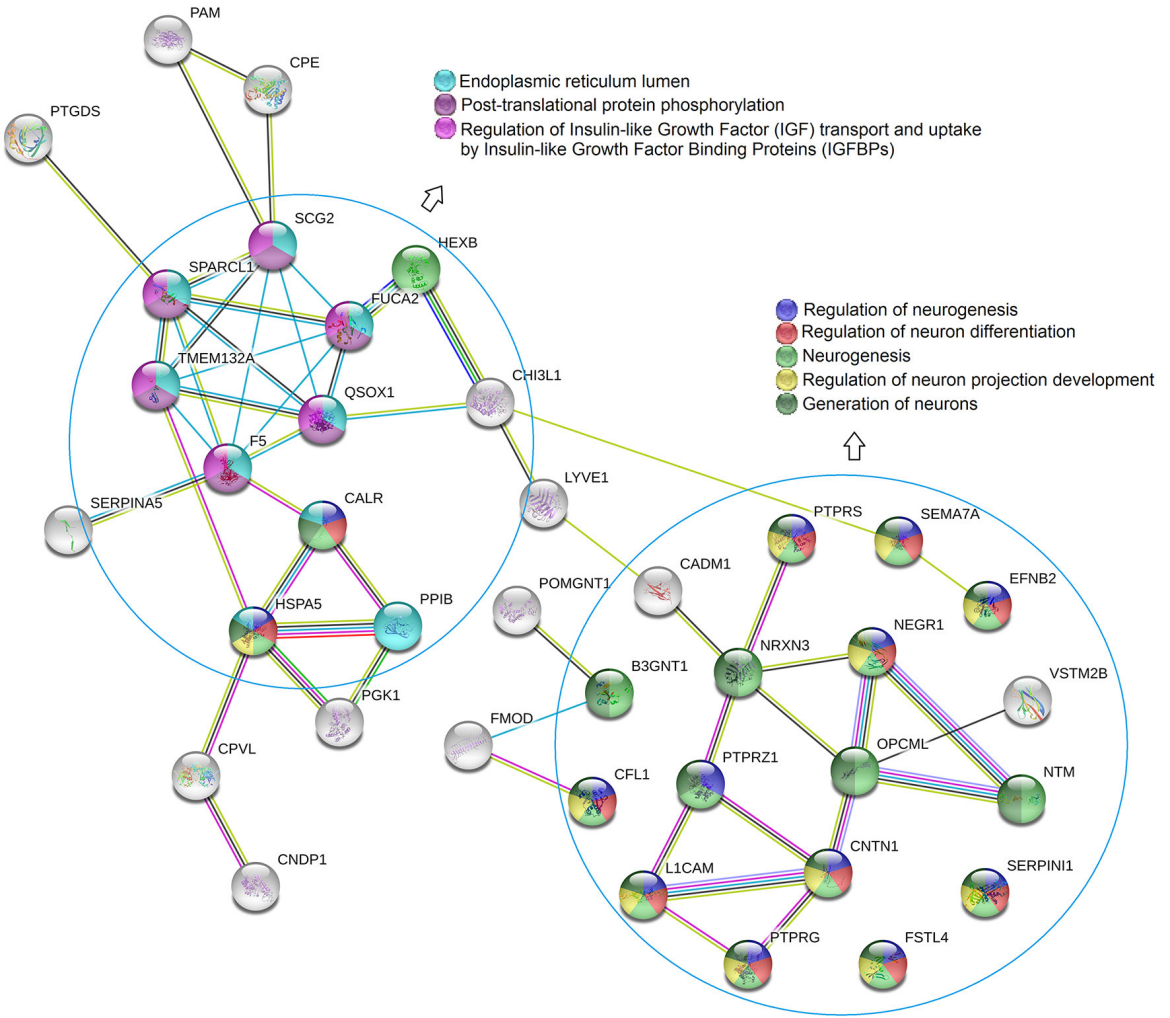
STRING protein-protein interaction diagram. GO and pathway enrichment analysis revealed that a cluster of proteins were involved in biological processes of neurogenesis, neuron differentiation, and neuro project development. Another cluster of proteins which were located in endoplasmic reticulum lumen, were involved in the pathways of post-translational protein phosphorylation, regulation of insulin-like growth factor (IGF) transport and uptake by insulin-like growth factor binding proteins (IGFBPs).

**Figure 4:**
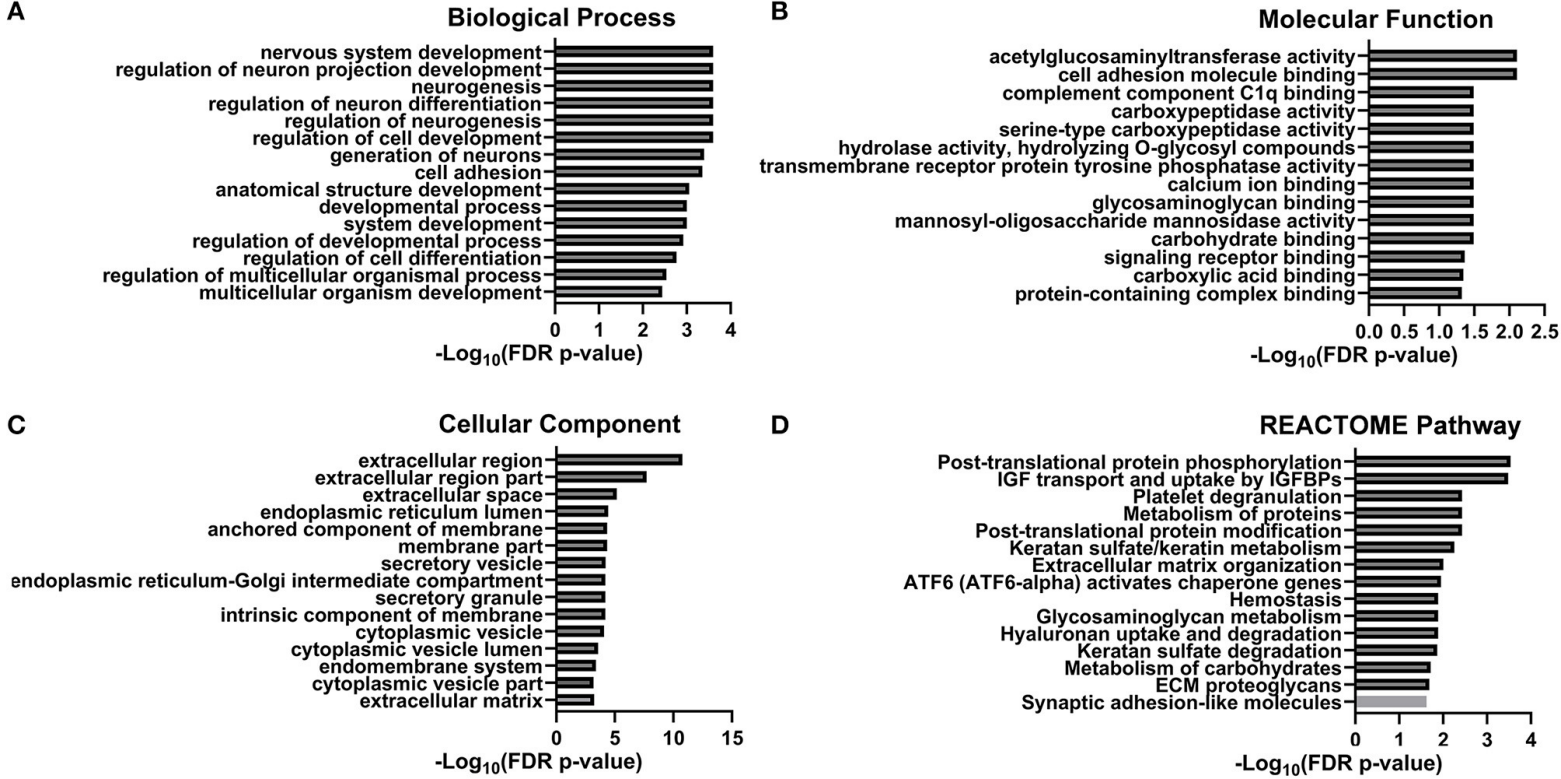
GO/KEGG column graph. The top 15 enriched items of the enrichment analysis including three GO categories [Biological Process **(A)**, Molecular Function **(B)**, and Cellular Component **(C)**] and regulative pathway [REACTOME Pathway **(D)**].

To further investigate potential pathological and biological mechanisms relevant to delirium, we performed bioinformatic analysis. The constructed protein–protein interaction network is shown in Figure 3. Thirty-eight proteins were included in this network, with known connections. GO and pathway enrichment analyses revealed a cluster of proteins that are involved in the biological processes of neurogenesis, neuron differentiation, and neuron projection development (B3GNT1, CFL1, PTPRS, NRXN3, PTPRZ1, L1CAM, SEMA7A, EFNB2, NEGR1, OPCML, CNTN1, PTPRG, NTM, SERPINI1, FSTL4, HEXB, CALR, and HSPA5). Another cluster of proteins (CALR, F5, FUCA2, HSPA5, PPIB, QSOX1, SCG2, SPARCL1, and TMEM132A), which are all located in the endoplasmic reticulum lumen, are involved in pathways of post-translational protein phosphorylation, regulation of insulin-like growth factor (IGF) transport, and uptake by IGF binding proteins (IGFBPs). The top 15 enriched items from the enrichment analysis included three GO categories (biological process, molecular function, and cellular component), and regulatory pathways (REACTOME database) were also shown (Figure 4, Table S2).

### Correlation Analysis

We assessed the relationship between MDAS scores, which reflect the severity of POD, and the levels of the 63 proteins that were abnormally expressed in CSF. The data were fit using a Pearson correlation analysis. The preoperative CSF levels of V-set and transmembrane domain-containing protein 2B (VSTM2B) and coagulation factor V (FA5) correlated with MDAS scores on postoperative day 1, and preoperative VSTM2B also correlated with MDAS scores on postoperative day 2 (*r* > 0.8, *p* < 0.05; Table 3).

**Table 3:** Correlation between MDAS scores and the preoperative CSF level of proteins.

**Proteins**	**MDAS score (postoperative day 1)**		**MDAS score (postoperative day 2)**	
	**Correlation coefficient r**	***P*-value**	**Correlation coefficient r**	***P*-value**
VSTM2B	0.869	0.005	0.928	0.008
FA5	0.840	0.002	0.636	0.090
RGMB	−0.711	0.032	−0.091	0.846
PXDC2	0.669	0.034	0.735	0.038

*MDAS, Memorial Delirium Assessment Scale; CSF, cerebrospinal fluid; VSTM2B, V-set and transmembrane domain-containing protein 2B; FA5, coagulation factor V; RGMB, repulsive guidance molecule domain family member B; PXDC2, Plexin domain-containing protein 2*.

## Discussion

Previous CSF proteomic studies in older orthopedic patients with delirium have been reported. The first was conducted by Poljak et al. (17) they performed an iTRAQ-based quantitative proteomic analysis of CSF, but their sample size was small (five POD patients and eight No-POD patients). In their study, seven protein expression levels were significantly dysregulated in at least three out of five POD subjects (17). The other such study was reported by Westhoff et al. (18). In their study, dysregulated proteins were found using two-dimensional fluorescence differences in gel electrophoresis and then identified by tandem mass spectrometry; the technique that was used is now obsolete (18). In the present study, we used LC-MS-based untargeted proteomic techniques to screen CSF protein expression profiles and compare the results from POD and No-POD patients. PRM-based targeted proteomic analysis was used to validate our results. Combined with bioinformatic analysis, our results give a comprehensive view of dysregulated protein profiles in the CSF of POD patients, providing valuable information for further biomarker screening and pathological mechanism studies.

In the untargeted proteomic results, there was no obvious difference in the protein expression of albumin (fold change = 1.05, *p* = 0.65), which is the most highly expressed protein in the serum and CSF, suggesting that total protein concentration might be not significantly different in POD patients. Most of the proteins that were dysregulated in the present study showed trends of downregulation in POD patients, which suggests that low concentrations of specific CSF proteins that are important in the nervous system might be involved in POD pathology.

To further retrieve biological information from the proteomic data, we employed bioinformatic analysis, using the 63 dysregulated proteins as inputs. In the constructed protein–protein interaction network combined with GO annotation, two clusters were found that were closely related to the nervous system (Figures 3, 4). One cluster contained proteins that are involved in neurogenesis as well as neuron generation, differentiation, and projection development, suggesting that a possible mechanism underlying POD onset may involve neuronal dysfunction. For example, neural cell adhesion molecule L1 (L1CAM), which is most highly expressed in the right hemisphere of the cerebellum, is involved in the dynamics of cell adhesion, neuronal migration, axonal growth, and synaptogenesis. In the present study, the preoperative concentrations of L1CAM in the CSF of patients with POD were significantly lower than those of patients without POD (fold change POD/No-POD = 0.579, *p* = 0.018). Increased levels of L1CAM might therefore be beneficial for older orthopedic patients, and this is similar to the idea that exogenous expression of L1CAM is beneficial in Alzheimer's disease. Djogo et al. reported that L1CAM overexpression in neurons and astrocytes improved histopathological and biochemical changes underlying Alzheimer's disease in an animal model (19). They proposed that the beneficial effects of L1CAM were mediated via multiple mechanisms, including their novel finding that L1CAM binds specifically to Aβ and reduces Aβ aggregation, as well as its previously described neurotrophic effect (20) and reduction in astrocyte activation (21). Our results encourage investigations into other effects of L1CAM, and may lead to the design of clinically viable ways to apply compounds that encompass L1CAM functions or antibodies that trigger L1CAM function.

The other cluster that we identified in the constructed protein–protein interaction network (Figures 3, 4) consisted of proteins located in the endoplasmic reticulum lumen that have protein phosphorylation and IGF pathway regulation functions; this finding provides clues to a possible mechanism by which neurons become dysfunctional in POD. One of the proteins in cluster two was FA5, which is the central regulator of hemostasis. Although there are no previous studies of the relationship between FA5 and POD, FA5 is the most commonly studied prothrombotic gene linked with ischemic stroke, which is a complex multifactorial neurological disorder. In the current study, the preoperative CSF concentrations of FA5 were significantly lower in patients with POD than in patients without POD (fold change POD/No-POD = 0.750, *p* = 0.007). In addition, preoperative CSF levels of FA5 positively correlated with MDAS scores on postoperative day 1 (correlation coefficient *r* = 0.840, *p* = 0.002), but not on postoperative day 2 (correlation coefficient *r* = 0.627, *p* = 0.096). However, the *p*-value for the correlation between preoperative CSF FA5 levels and postoperative day 2 MDAS scores was close to 0.05; in a larger sample size this correlation might therefore reach significance. In a previous study using a rat model of menopause, 17β-estradiol replacement improved hippocampus-dependent spatial memory and caused robust activation (fold change > 10) of hippocampal *FA5* gene expression (22). FA5 is the most prevalent genetic variation that leads to prothrombotic state; this gene is therefore considered important for understanding the mechanisms of stroke (23). Another previous study revealed that *FA5* polymorphisms confer an increased risk of ischemic stroke in younger adults (24). Furthermore, Bhattacharjee et al. reported that the FA5 activator might be associated with Alzheimer's disease; the FA5 activator destabilized Aβ aggregates, which may be useful for disease prevention in the future (25). An earlier study found that the risk of dementia increased 2.11-fold in carriers of the *FA5* Leiden mutation relative to subjects lacking this mutation [95% confidence interval [CI] 0.93–4.77]. The increased risks of vascular dementia and Alzheimer's disease were 4.28 (95% CI 1.26–14.5) and 2.15 (95% CI 0.82–5.63), respectively (26). Thus, the relationship between FA5 and POD requires further study.

Another protein that was dysregulated in POD patients in the current study is VSTM2B, a 285-amino-acid, single-pass, type I membrane protein containing an Ig-like V-type (immunoglobulin-like) domain. The preoperative CSF concentrations of VSTM2B were significantly lower in patients with POD than in those without POD (fold change POD/No-POD = 0.558, *p* = 0.032). In addition, there was a significant correlation between preoperative CSF VSTM2B levels and POD severity. Preoperative VSTM2B positively correlated with MDAS scores on both postoperative day 1 (correlation coefficient *r* = 0.869, *p* = 0.005) and day 2 (correlation coefficient *r* = 0.928, *p* = 0.008). A previous study investigated differentially expressed genes in the dorsal root ganglia in a neuropathic pain from spared nerve injury model; they reported that VSTM2B was downregulated, which indicates that reduced VSTM2B is related to neuropathic pain (27). Another recent study used proteomics to identify CSF biomarkers in genetic frontotemporal dementia, and found that symptomatic granulin mutation carriers had significantly lower levels of VSTM2B than both non-carriers and presymptomatic carriers (28). The exact function of VSTM2B has not previously been studied in detail, and as yet there are no literature reports on the relationship between VSTM2B and POD. Further validation is therefore needed to investigate the clinical applicability of VSTM2B as a diagnostic or monitoring biomarker of POD.

Our study has some limitations. In this cohort study, no power calculations were performed. Instead, we aimed to recruit the maximum number of patients possible with the resources available, which was 80 patients. We included 10 POD patients and 30 NOPOD patients in the discovery stage, and 5 POD patients were later included in the validation stage. Further validation analysis needs to be performed in larger cohorts in future studies.

In conclusion, we identified and validated several novel dysregulated CSF proteins at greater risk regarding in POD, and revealed several pathways relevant to POD development. Our results not only provide potential risk biomarkers for POD, but also give clues for further investigations into the pathological mechanisms of delirium. Strengthen research on potential biomarkers through peripheral body fluid (blood, urine, et al.), we believe that they have translational impact on clinical prediction of POD.

## Data Availability Statement

All datasets generated for this study are included in the article/Supplementary Material.

## Ethics Statement

The studies involving human participants were reviewed and approved by Beijing Jishuitan Hospital Medical Science Research Ethics Committee (JLKS201901-04). The patients/participants provided their written informed consent to participate in this study.

## Author Contributions

YH, WC, ML, JZ, and XG designed the study. YH, YS, YY, TL, DH, and XM collected samples and performed clinical-related analyses. WC and LZ performed proteomic experiments. JZ analyzed the data. YH, ZL, and YZ reviewed statistical analyses. YH and JZ wrote the manuscript. All authors read and approved the final manuscript.

### Conflict of Interest

The authors declare that the research was conducted in the absence of any commercial or financial relationships that could be construed as a potential conflict of interest.
