# Supplementary material for: Proteomic Analysis of Preoperative CSF Reveals Risk Biomarkers of Postoperative Delirium
Source: Front Psychiatry. 2020 Mar 4;11:170. doi: 10.3389/fpsyt.2020.00170 (PMC7064445; doi:10.3389/fpsyt.2020.00170)
Supplement: Supplementary file 1 [file Table_1.DOCX]

**Table S1. Dysregulated proteins revealed by untargeted proteomics.**

| Protein ID | Fold change (POD/No-POD) | *P* Value |
| --- | --- | --- |
| sp\|Q5BLP8\|CD048_HUMAN | 0.525188157 | 0.0129285 |
| sp\|Q8TAG5\|VTM2A_HUMAN | 0.552590618 | 0.0380422 |
| sp\|A6NLU5\|VTM2B_HUMAN | 0.558393256 | 0.0318223 |
| sp\|Q8IUK5\|PLDX1_HUMAN | 0.561563349 | 0.0211786 |
| sp\|P48058\|GRIA4_HUMAN | 0.573578535 | 0.0099322 |
| sp\|P32004\|L1CAM_HUMAN | 0.579262871 | 0.0176409 |
| sp\|Q06828\|FMOD_HUMAN | 0.62322109 | 0.030122 |
| sp\|Q02487\|DSC2_HUMAN | 0.640778561 | 0.0175197 |
| sp\|Q6MZW2\|FSTL4_HUMAN | 0.642185658 | 0.0308302 |
| sp\|P07711\|CATL1_HUMAN | 0.657097266 | 0.0007754 |
| sp\|P52799\|EFNB2_HUMAN | 0.669219528 | 0.0233388 |
| sp\|Q6NW40\|RGMB_HUMAN | 0.670039775 | 0.0224965 |
| sp\|O95967\|FBLN4_HUMAN | 0.68471393 | 0.0394013 |
| sp\|Q8WZA1\|PMGT1_HUMAN | 0.692540684 | 0.0041811 |
| sp\|Q24JP5\|T132A_HUMAN | 0.696427758 | 0.0191645 |
| sp\|P23284\|PPIB_HUMAN | 0.704945059 | 0.0100657 |
| sp\|Q9Y5Y7\|LYVE1_HUMAN | 0.706014726 | 0.0301129 |
| sp\|O95502\|NPTXR_HUMAN | 0.716105332 | 0.0473163 |
| sp\|P22105\|TENX_HUMAN | 0.719854431 | 0.0243064 |
| sp\|P23470\|PTPRG_HUMAN | 0.720563434 | 0.0256475 |
| sp\|Q6UXD5\|SE6L2_HUMAN | 0.721271666 | 0.0190466 |
| sp\|Q7Z3B1\|NEGR1_HUMAN | 0.72304904 | 0.0281246 |
| sp\|Q15904\|VAS1_HUMAN | 0.724069893 | 0.0118521 |
| sp\|Q9Y4C0\|NRX3A_HUMAN | 0.727398094 | 0.0172686 |
| sp\|Q14982\|OPCM_HUMAN | 0.730757688 | 0.026178 |
| sp\|Q9UBQ6\|EXTL2_HUMAN | 0.731156853 | 0.0108952 |
| sp\|Q9BY67\|CADM1_HUMAN | 0.731809747 | 0.0172612 |
| sp\|O75144\|ICOSL_HUMAN | 0.733194642 | 0.0212364 |
| sp\|Q9H3G5\|CPVL_HUMAN | 0.734279851 | 0.0209928 |
| sp\|O43505\|B4GA1_HUMAN | 0.735134997 | 0.0117822 |
| sp\|Q99574\|NEUS_HUMAN | 0.742096595 | 0.0455491 |
| sp\|O75326\|SEM7A_HUMAN | 0.74334133 | 0.0411907 |
| sp\|P49641\|MA2A2_HUMAN | 0.746036692 | 0.0228003 |
| sp\|P12259\|FA5_HUMAN | 0.750377347 | 0.0074559 |
| sp\|P33908\|MA1A1_HUMAN | 0.751074198 | 0.0365002 |
| sp\|P36222\|CH3L1_HUMAN | 0.751932565 | 0.0193597 |
| sp\|P07686\|HEXB_HUMAN | 0.752386382 | 0.0153197 |
| sp\|P23471\|PTPRZ_HUMAN | 0.755306035 | 0.0146818 |
| sp\|P43251\|BTD_HUMAN | 0.758254861 | 0.0024663 |
| sp\|P05154\|IPSP_HUMAN | 0.764390003 | 0.0200905 |
| sp\|Q13332\|PTPRS_HUMAN | 0.771624843 | 0.0180858 |
| sp\|P26992\|CNTFR_HUMAN | 0.772774907 | 0.0398741 |
| sp\|P19021\|AMD_HUMAN | 0.773400056 | 0.0311183 |
| sp\|Q9P121\|NTRI_HUMAN | 0.777165712 | 0.0354655 |
| sp\|P16870\|CBPE_HUMAN | 0.777413485 | 0.0394 |
| sp\|Q14515\|SPRL1_HUMAN | 0.779929723 | 0.00805 |
| sp\|P13521\|SCG2_HUMAN | 0.780583466 | 0.0402299 |
| sp\|P20062\|TCO2_HUMAN | 0.780872973 | 0.0462374 |
| sp\|Q9BTY2\|FUCO2_HUMAN | 0.791548673 | 0.034448 |
| sp\|Q6UX73\|CP089_HUMAN | 0.79160734 | 0.0262792 |
| sp\|P11021\|BIP_HUMAN | 0.792299978 | 0.0064205 |
| sp\|Q96KN2\|CNDP1_HUMAN | 0.795796946 | 0.0360522 |
| sp\|O00391\|QSOX1_HUMAN | 0.79957704 | 0.0093825 |
| sp\|P49908\|SEPP1_HUMAN | 0.804159789 | 0.0233985 |
| sp\|Q6UX71\|PXDC2_HUMAN | 0.815847872 | 0.0354194 |
| sp\|P27797\|CALR_HUMAN | 0.820135986 | 0.0123648 |
| sp\|Q12860\|CNTN1_HUMAN | 0.822361147 | 0.0326568 |
| sp\|P41222\|PTGDS_HUMAN | 0.887585392 | 0.0351564 |
| sp\|P01857\|IGHG1_HUMAN | 1.370887154 | 0.0415034 |
| sp\|P00558\|PGK1_HUMAN | 2.034815028 | 0.0292314 |
| sp\|P23528\|COF1_HUMAN | 2.543209896 | 0.0384416 |
| sp\|P00915\|CAH1_HUMAN | 5.17676762 | 0.0409388 |
| sp\|P02042\|HBD_HUMAN | 6.033869257 | 0.0277123 |
