# Supplementary material for: Proteomic Analysis of Preoperative CSF Reveals Risk Biomarkers of Postoperative Delirium
Source: Front Psychiatry. 2020 Mar 4;11:170. doi: 10.3389/fpsyt.2020.00170 (PMC7064445; doi:10.3389/fpsyt.2020.00170)
Supplement: Supplementary file 2 [file Table_2.DOCX]

**Table S2. GO and pathway enrichment results.**

| Protein ID | Description | Observed  gene | Background gene | False discovery rate | Matching proteins |
| --- | --- | --- | --- | --- | --- |
| HSA-8957275 | Post-translational protein phosphorylation | 6 | 106 | 0.00029 | F5,FUCA2,QSOX1,SCG2,  SPARCL1,TMEM132A |
| HSA-381426 | Regulation of Insulin-like Growth Factor (IGF) transport and uptake by Insulin-like | 6 | 123 | 0.00033 | F5,FUCA2,QSOX1,SCG2,  SPARCL1,TMEM132A |
| HSA-114608 | Platelet degranulation | 5 | 125 | 0.0037 | CFL1,F5,HSPA5,QSOX1,  SEPP1 |
| HSA-392499 | Metabolism of proteins | 17 | 1948 | 0.0037 | B3GNT1,CALR,CPE,  EXTL2,F5,FUCA2,HSPA5,MAN1A1,MAN2A2,  NEGR1,NTM,OPCML,  POMGNT1,QSOX1,SCG2,  SPARCL1,TMEM132A |
| HSA-597592 | Post-translational protein modification | 14 | 1366 | 0.0037 | B3GNT1,CALR,F5,FUCA2,MAN1A1,MAN2A2,  NEGR1,NTM,OPCML,POMGNT1,QSOX1,SCG2,  SPARCL1,TMEM132A |
| HSA-1638074 | Keratan sulfate/keratin metabolism | 3 | 33 | 0.0055 | B3GNT1,FMOD,HEXB |
| HSA-1474244 | Extracellular matrix organization | 6 | 298 | 0.0098 | CTSL,EFEMP2,FMOD,  PPIB,PTPRS,TNXB |
| HSA-381183 | ATF6 (ATF6-alpha) activates chaperone genes | 2 | 9 | 0.0112 | CALR,HSPA5 |
| HSA-109582 | Hemostasis | 8 | 601 | 0.013 | CFL1,F5,HBD,HSPA5,  L1CAM,QSOX1,SEPP1,  SERPINA5 |
| HSA-1630316 | Glycosaminoglycan metabolism | 4 | 122 | 0.013 | B3GNT1,FMOD,HEXB,  LYVE1 |
| HSA-2160916 | Hyaluronan uptake and degradation | 2 | 12 | 0.013 | HEXB,LYVE1 |
| HSA-2022857 | Keratan sulfate degradation | 2 | 13 | 0.0136 | FMOD,HEXB |
| HSA-71387 | Metabolism of carbohydrates | 5 | 266 | 0.0192 | B3GNT1,FMOD,HEXB,  LYVE1,PGK1 |
| HSA-3000178 | ECM proteoglycans | 3 | 75 | 0.0206 | FMOD,PTPRS,TNXB |
| HSA-8849932 | Synaptic adhesion-like molecules | 2 | 21 | 0.0238 | GRIA4,PTPRS |
| HSA-140875 | Common Pathway of Fibrin Clot Formation | 2 | 22 | 0.0246 | F5,SERPINA5 |
| HSA-3296482 | Defects in vitamin and cofactor metabolism | 2 | 22 | 0.0246 | BTD,TCN2 |
| HSA-6794362 | Protein-protein interactions at synapses | 3 | 85 | 0.0246 | GRIA4,NRXN3,PTPRS |
| HSA-983170 | Antigen Presentation: Folding, assembly and peptide loading of class I MHC | 2 | 25 | 0.027 | CALR,HSPA5 |
| HSA-163125 | Post-translational modification: synthesis of GPI-anchored proteins | 3 | 92 | 0.0271 | NEGR1,NTM,OPCML |
| HSA-2022854 | Keratan sulfate biosynthesis | 2 | 27 | 0.0276 | B3GNT1,FMOD |
| HSA-381119 | Unfolded Protein Response (UPR) | 3 | 94 | 0.0276 | CALR,EXTL2,HSPA5 |
| HSA-8950505 | Gene and protein expression by JAK-STAT signaling after Interleukin-12 stimulation | 2 | 38 | 0.0498 | CA1,CFL1 |
